# Family support and ease of access link socio-economic status and sports club membership in adolescent girls: a mediation study

**DOI:** 10.1186/1479-5868-10-50

**Published:** 2013-04-25

**Authors:** Rochelle M Eime, Jack T Harvey, Melinda J Craike, Caroline M Symons, Warren R Payne

**Affiliations:** 1 Institute of Sport, Exercise and Active Living, Victoria University, PO Box 14428, Melbourne, Victoria 8001, Australia; 2 School of Health Sciences, University of Ballarat, PO Box 663, Ballarat, Victoria 3353, Australia; 3 Faculty of Health, Deakin University, 221 Burwood Hwy, Burwood, Victoria, 3125, Australia

**Keywords:** Socio-economic status, Club sport, Adolescent females, Family support, Access

## Abstract

**Background:**

Much research has been conducted into the determinants of physical activity (PA) participation among adolescent girls. However, the more specific question of what are the determinants of particular forms of PA participation, such as the link between participation through a sports club, has not been investigated. Accordingly, the aim of this study was to investigate the relationships between participation in a sports club and socio-economic status (SES), access to facilities, and family and peer support, for female adolescents.

**Methods:**

A survey of 732 female adolescent school students (521 metropolitan, 211 non-metropolitan; 489 Year 7, 243 Year 11) was conducted. The survey included demographic information (living arrangements, ethnicity indicators, and indicators of SES such as parental education and employment status and locality); access to facilities; and family and peer support (travel, encouragement, watching, praise, joint participation). For each characteristic, sports club participants and non-participants were compared using chi-square tests. Multiple mediation analyses were used to investigate the role of access, family and peer support in the link between SES and sport participation.

**Results:**

There were significant associations (p<0.05) between sports club participation and: all demographic characteristics; all measures of family and peer support; and access to sport-related facilities. Highest levels of participation were associated with monolingual Australian-born families, with two parents, at least one of whom was well-educated, with both parents employed, and high levels of parental assistance, engagement and support. Participation in club sport among both younger and older adolescent girls was significantly positively associated with the SES of both their neighbourhoods and their households, particularly in metropolitan areas. These associations were most strongly mediated by family support and by access to facilities.

**Conclusions:**

To facilitate and promote greater participation in club sport among adolescent girls from low SES neighbourhoods and households, strategies should target modifiable determinants such as facility access and parental support. This will involve improving access to sports facilities and promoting, encouraging and assisting parents to provide support for their daughters’ participation in sport clubs.

## Background

The increasing prevalence of overweight and obesity in many countries has been shown to contribute to the development of a range of chronic diseases and is considered by many to constitute a public health crisis. Limited physical activity (PA) is acknowledged as a major factor contributing to the development of this crisis [1]. It is also documented that participation in PA declines during adolescence, particularly for females [2,3].

There is substantial literature describing the determinants of participation in PA for adolescents, and these are increasingly being understood using socio-ecological models [4,5]. It is now common practice to examine determinants across the different domains (intrapersonal, interpersonal, organisational/environmental) of socio-ecological models [6-9].

In socio-ecological terms, socio-economic status (SES) has aspects of the intrapersonal (e.g. education level, employment status and income of the individual), interpersonal (e.g. education level, employment status and income of parents or caregivers) and environmental (e.g. profiles of education level, employment status and income for neighbourhoods as a whole). Another important SES-related environmental factor is access to facilities, which in turn has various aspects including levels of provision (presence), geographical accessibility (proximity) and affordability.

SES is associated with PA participation, with those adolescents from higher SES households and/or neighbourhoods more likely to participate in PA [8,10-14]. There is evidence that adolescents whose parents have attained higher education levels are more likely to participate in organised sport, structured exercise and games play in their leisure time than those with parents with lower education levels [15]. However, research by Ball et al., 2009 reported no prospective associations between SES (maternal education) and objectively measured PA amongst children aged 5–6 and 10–12 [16]. With regard to employment, some recent research has indicated that adolescents with one or two unemployed parents were less likely to participate in sports that those with two employed parents [17].

Family support and support from peers/friends are key interpersonal factors which significantly influence adolescents’ PA behaviour [13,14,18-21], including participation in leisure-time sport [9]. However, there is inconclusive evidence regarding the changing nature of perceived family support across the transitional period of adolescence. It has been proposed that family support is more important for older adolescents [20], whilst other researchers have found no difference in the level of perceived parental support by age [21]. Research that has examined this influence by parental gender showed that fathers provide more encouragement than mothers, and that this support has a greater influence when adolescents are younger rather than older [22,23]. The nature of social support and its contribution to adolescents’ physical activity levels is complex, nonetheless most studies report family support as a critical factor [13,21].

Whilst we know that support from both family and peers/friends are strong determinants of PA behaviour, there is a growing body of literature suggesting that support from peers/friends is a stronger predictor of PA than support from parents [20,24-28]. In a recent qualitative study, older adolescent girls reported participating in community club sport primarily to be with their friends [29]. In a related study, younger adolescents reported participating in sport and PA to be involved with friends, with support from family and teachers providing role modelling and positive feedback which facilitated their participation [30].

Recent study results have indicated that of various psychosocial determinants, family social support was the strongest predictor of PA behaviour for female adolescents, and friend social support was not related [18]. However, the details of how the two support mechanisms of family and friends alter throughout the adolescent transition period, and how these intrapersonal factors interact with the environmental determinant ‘access’ are unclear.

Environmental determinants such as presence of and proximity to facilities have been investigated, with some finding that the simple presence of public facilities including sports facilities, is positively correlated with PA level [14,31]. Furthermore, proximity to these facilities is positively associated with their use [32]. However, as Tucker (2009) suggests, in addition to actual accessibility we need to consider the role that perceived accessibility has in determining the use of facilities [33]. Another issue relevant to adolescents, of which we need to have a better understanding, is parents’ willingness and capacity to provide or facilitate transport to PA activities [13].

Broader geographical differences between localities constitute another potential environmental determinant of PA which has not been extensively investigated. However, qualitative research has shown, that adolescents living in non-metropolitan (regional/rural) areas are more likely than metropolitan-living adolescents to report limited opportunities (facilities, types of activities, number of teams), proximity to facilities, and cost and availability of transport, as barriers to participation [27,29,30,34].

Research to date has given little attention to the determinants of different forms of PA, and has largely been focused either on general levels of total PA [21,35] or leisure time PA [36]. Sport is a popular form of PA amongst adolescents [10,37] and we know that involvement in club sport can impact positively on social and psychological well-being [38-41], with potentially greater physical and psychological health benefits than other forms of PA [42,43]. Adolescents have ranked their life satisfaction higher if they were involved with sports clubs [10]. Community sports clubs provide opportunities for social interaction through both structured (organised and competitive) and unstructured (social) participation in sport [44]. For example, clubs may work as social catalysts, leading to enhanced involvement and participation [45]. Participation in sport can also enhance social connectedness, social support, peer bonding, life satisfaction and self-esteem, and may reduce stress, anxiety and depression, [39,40,42,43,46]. As with PA in general, participation in organised sport declines in the older adolescent years [37,47,48], particularly for females [17]. However, notwithstanding the recognised benefits of sport, there has been little research into the determinants of sport participation per se, or into the factors influencing the decline in sport participation through the adolescent years. Furthermore, the level of physical activity, and more specifically participation in sport, is consistently lower for female adolescents than it is for males [49-52].

In summary, we are beginning to understand the key determinants of PA across the domains of the socio-ecological model. However, as various authors have acknowledged, the current literature does not provide insight into how intrapersonal, interpersonal and environmental determinants for adolescents differ according to different stages/ages or locations (metropolitan v. regional/rural) [14] or different family structures [53]. Furthermore, the determinants of PA in the important sport context are not well understood. Accordingly, the aim of this study was to investigate the relationships between participation in a sports club and SES, access to facilities, and family and peer support, for female adolescents in Year 7 and Year 11, living in metropolitan and regional/rural areas.

We hypothesised that sports club membership would be associated with a range of factors (potential determinants), including measures of household SES, other household demographic characteristics, and area SES. We further hypothesised that these associations would be mediated by access to facilities, family support and peer support. Finally, we aimed to examine if any of these relationships were moderated by school year- level or geographical setting.

## Methods

### Design

This research was based on a cross-sectional survey of female adolescents, recruited through their schools, using a self-completion questionnaire.

### Procedure

Schools in metropolitan, regional and rural areas of Victoria, Australia, were randomly selected and invited to participate. The postcodes of schools were used to assign a value of the Australian Bureau of Statistics (ABS) SEIFA (Socio-economic Indices for Areas) Index of Relative Socio-economic Advantage and Disadvantage (IRSAD) [54] to each, and the distribution of schools was checked to ensure they were representative of the broader IRSAD distribution in Victoria. A total of 17 schools in the metropolitan area (34% of 50 contacted) and 14 schools in rural and regional areas (88% of 16 contacted) participated in the study. Ethical approval was gained from the University Human Research Ethics Committees, the Victorian Department of Education and the Victorian Catholic Education Office.

A pilot test was conducted, involving 71 respondents in a convenience sample of three schools. Minor changes were made to the content of the questionnaire and the order of questions was revised.

During the southern hemisphere Autumn (April), all female students in years 7 and 11 of participating schools were invited (by the Physical Education coordinator or a researcher) to participate, and plain language information statements and parental and respondent consent forms were distributed. Students who returned both self and parent completed consent forms within the stipulated time completed the baseline questionnaire, usually during school class time. Consent rates were as follows: Metropolitan - Year 7 23.7% (368 of 1550 distributed), Year 11 13.0% (155 of 1189 distributed); Non-metropolitan - Year 7 25.7% (123 of 479 distributed), Year 11 16.8% (88 of 523 distributed).

### Measures

The questionnaire included questions regarding: demographic characteristics of participants and their parents/caregivers, including household structure, education, employment, ethnicity, religion, sport and PA participation; and potential determinants of sport and PA participation.

Derived variables used in this study were as follows. The dependent variable (DV) was *current membership of a sports club* (dichotomous self-report: yes/no) Sports club membership may involve competitive and/or recreational participation, although competition is the predominant form of activity provided by sports clubs in Australia. The explanatory variables (EVs) were three SES indicators: *highest educational qualification of parent(s)/caregiver(s)* (dichotomous: sole or both less than Year 12 v at least one Year 12 and above); *employment status of parent(s)/caregiver(s)* (dichotomous: two parents/caregivers both in F/T or P/T employment v lower levels of employment); *and SES of home postcode* (Socio-economic Indices for Areas (SEIFA) Index of Relative Socio-economic Advantage and Disadvantage (IRSAD) [54]. The IRSAD index scores were categorised into quintiles (5 categories with approximately equal frequencies) for chi-square analysis, and transformed into standardised z-scores for mediation analysis. Potential mediators were: *access* to a range of sport and PA facilities; *family support* for participation in sport and PA; and *peer support* for participation in sport and PA [55]. Each potential mediator variable was derived from on a set of Likert-scale items (see Table 1). The response categories were as follows: *access*- I don’t know where one is; I can’t get there; I can get there, but only if an adult provides transport; I can get there by myself (walking or by bike or public transport); *family support* and *peer support*- never; hardly ever; sometimes; often; all the time. In each case a summated scale was derived, using principal components analysis and inter-item reliability analysis.

**Table 1:** **Results**^**1 **^**of chi**-**square tests of association between sports club membership and potential determinants and mediators**

		**All**	**Sub**-**samples**				**Category with**
**Aspect**	**Variable**	**cases *N*=710**^**2**^	**Metro *n*=510**^**2**^	**Rur/Reg *n*=200**^**2**^	**Year 7 *n*=470**^**2**^	**Year 11 *n*=240**^**2**^	**highest rate of sports club membership (all cases)**
Household	Household Structure	**	NS	*	**	NS	Two parents ± siblings
Ethnicity	Ethnicity of family	***	***	NS	***	***	All born in Australia
	Languages other than English spoken at home	***	***	**	***	***	No other languages spoken
Religion	Religion	***	**	**	**	NS	Christian or no religion
SES	SEIFA IRSAD index quintiles	***	***	NS	**	***	Highest quintile
	Employment status of parents	***	**	NS	**	NS	Two parents employed F/T or P/T
	Education levels of parents	**	**	NS	*	NS	Degree
Access	Aerobic/dance	***	***	NS	***	NS	More accessible
	Athletics track	***	***	*	***	**	More accessible
	Beach	NS	NS	NS	*	NS	
	Bike lanes on roads	NS	*	NS	*	NS	
	Courts (eg tennis)	**	*	NS	*	**	More accessible
	Fitness centre / gym	NS	NS	NS	*	NS	
	Golf course	***	**	**	**	**	More accessible
	Lake, river, creek, dam	NS	NS	NS	NS	NS	
	Martial arts studio	NS	NS	NS	NS	NS	
	Park / playground	NS	NS	*	NS	NS	
	Playing field (eg soccer)	***	***	**	***	**	More accessible
	Sporting goods store	***	*	*	***	NS	More accessible
	Swimming pool	***	**	NS	**	NS	More accessible
	Walking/running/cycling track	NS	*	NS	NS	NS	
Family	Willing to assist you to travel?	***	***	***	***	***	All the time
support	Encouraged you to do PA or sport?	***	***	***	***	***	All the time
	Done a PA or sport with you?	***	***	***	***	***	All the time
	Watched you participate in PA or sport?	***	***	***	***	***	All the time
	Told you that you are doing well in PA or sport?	***	***	***	***	***	All the time
Peer	Do you encourage your friends to do PA or sport?	***	***	***	***	***	All the time
support	Do your friends encourage your to do PA or sport?	***	**	**	***	*	Often
	Do your friends do PA or sport with you?	***	***	***	***	***	All the time
	Do other kids tease you for not being good at PA or sport?	**	**	NS	**	NS	Never
	Do friends tell you that you are doing well in PA or sport?	***	**	**	***	**	All the time

^1^ NS Not significant * p<0.05 ** p<0.01 *** p<0.001 ^2^ Sample sizes are nominal; exact sample sizes for each variable were reduced by missing values due to non-response.

### Analyses

Pearson chi-square analyses were used to test the association between sports club membership and the three SES indicators, the other demographic characteristics, and each of the individual items from which the potential mediators were derived. Multiple mediation analyses utilising linear and logistic regression models as appropriate were used to further investigate the extent to which the association between sports club membership and each of the three SES indicators was mediated by the three potential mediators. In each case, the moderating effects of year level and region were also investigated by conducting separate analyses for four subsamples of the data (univariate “slices” for Year 7, Year 11, metropolitan, regional/rural). Data screening was undertaken prior to the data analyses. All analyses were conducted using SPSS Version 19 on the cases with complete data for the particular analysis.

## Results

### Participants

A total of 732 respondents completed the questionnaire. Of these 71.2% (n=521) were from metropolitan schools and 28.8% (n=211) were from rural and regional schools. Year 7 respondents accounted for 66.8% (n=489) of the sample and 33.2% (n=243) were from Year 11. The age range of respondents was 11–20 years (M=13.6, SD=1.96, n=701). The ages were clustered in the ranges 11–13 years (Year 7) and 16–20 years (Year 11).

Four hundred and fifty eight (63.0% of 727 respondents) were current members of a sports club. There were significant differences in club membership rates between year levels (Year 7 67.6%, Year 11 53.7%, p<0.001) and regions (metropolitan 58.6%, rural and regional 73.6%, p<0.001).

### Preliminary analysis

Table 1 shows results of chi-square tests of association between sports club membership and the potential determinants and potential mediators.

The first column of chi-square test results in Table 1 shows that sports club membership was significantly associated with all potential determinants and potential mediators except for access to the types of facility that are widespread, freely accessible and not associated with sport. The last column in Table 1 indicates which category of each potential determinant or mediator was associated with the highest proportion of sports club members. In general, a high incidence of sports club membership was associated with high SES (employment, education, SEIFA IRSAD), “traditional” nuclear family household structure, “mainstream” ethnicity and religion, good access to facilities and strong family and peer support. There were some variations in this pattern among the four subsamples. These variations are discussed under “moderation analysis” below.

### Mediation analysis

**Figure 1:**
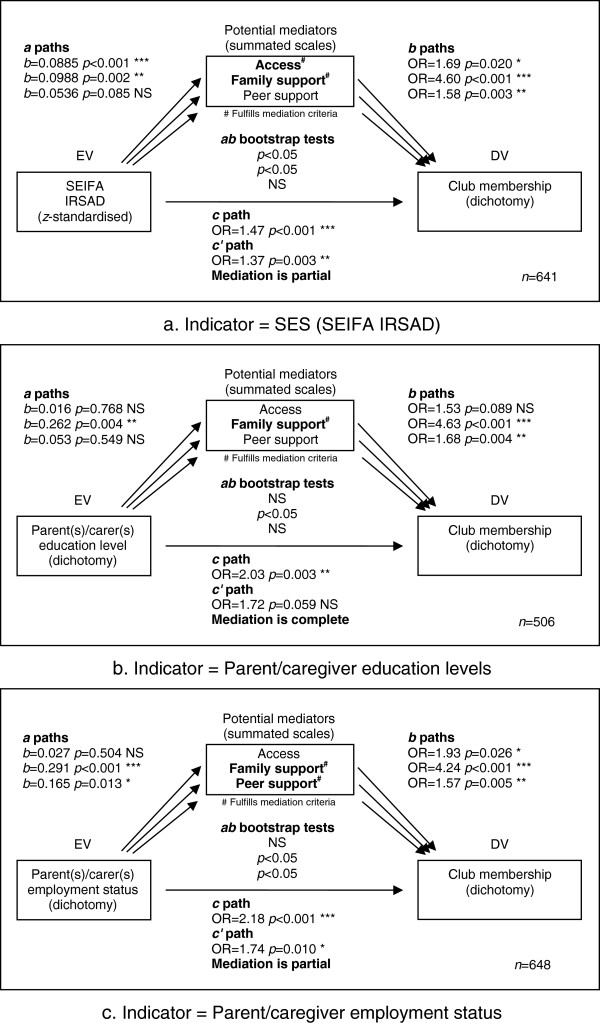
**Multiple mediation analyses of the association between sports club membership and three SES indicators. a**. Indicator = SES (SEIFA IRSAD). **b**. Indicator = Parent/caregiver education levels. **c**. Indicator = Parent/caregiver employment status.

To further investigate the significant associations between sports club membership and each of the three SES indicators, three multiple mediation analyses were conducted. To test the mediating effects of access to facilities, family support and peer support, the following sequence of regression models was evaluated (as outlined by [56]); (i) sports club membership was predicted from SES, using bivariate logistic regression (LR); (ii) access to facilities, family support and peer support were (separately) predicted from SES, using bivariate ordinary least squares (OLS) regression; (iii) sports club membership was predicted (in a four-predictor multiple LR model) from SES, access to facilities, family support and peer support. To establish mediation, SES must affect sports club membership (the “*c*” path in Figure 1) and at least one of access to facilities, family support and peer support (the “*a*” paths in Figure 1), which in turn must affect sports club membership (the “*b*” paths in Figure 1), and the signs of all the relationships must be in the expected direction. Furthermore, the effect of SES on sports club membership must be significantly reduced in the four-predictor model including access to facilities, family support and peer support (the “*c′*” path), compared with the one-predictor model with SES alone (the *c* path). In a simple mediation model, the difference c-*c*′ is equivalent to the product of *a* and *b* path coefficients, designated *ab*; in a multiple mediation model, c-*c*′ can be decomposed into separate *ab* terms for each mediator. The *ab* terms are tested using bias-corrected and accelerated bootstrapping [57] which results in a 95% confidence interval and hence an implicit significance test outcome rather than an explicit significance test with a *p*-value. If the *c*′ path remains significant, the mediation is said to be partial; if the *c*′ path is not significant, the mediation is said to be complete. To quantify the relative contribution of each mediator, a measure of effect size is the mediation ratio or relative indirect effect *ab/c*[58], which can be loosely interpreted as the proportion of the relationship accounted for by each mediator.

Figure 1 shows the results of the mediation analyses. Bivariate OLS regression coefficients (*b*) are shown for each *a* path, and odds ratios (OR) based on multiple and bivariate LR models respectively are shown for *b* and *c* paths. Results of bootstrap tests for *ab* (i.e. the *c*′-*c* difference) are also shown. In each case, the potential mediators meeting all mediation criteria are shown in boldface.

Figure 1 shows that sports club membership was significantly associated with higher levels of all three SES measures (*c* paths). Sports club membership was also significantly positively associated with all three potential mediators (*b* paths). The weakest link in the mediation chain was generally the *a* path, relating the SES measure to the potential mediator.

With regard to each SES indicator in turn, the significant association between SEIFA IRSD and sports club membership was partially mediated by access to facilities and family support, but not mediated by peer support. The significant association between parent(s)/carer(s) education level and sports club membership was completely mediated by family support, but not mediated by access to facilities or peer support. The significant association between parent(s)/carer(s) employment level and sports club membership was partially mediated by family support and peer support, but not mediated by access to facilities.

With regard to each mediator in turn, family support fulfilled all mediation criteria (significant association with SES and a significant indirect (mediated) effect of SES on sports club membership) for all three SES measures. Access to facilities mediated the association with SEIFA IRSAD only, and peer support mediated the association with parent/caregiver employment only.

The effect sizes (mediation ratios) were as follows: for SEIFA IRSD – facilities .12, family support .39, peer support .07; for parent(s)/carer(s) education level – facilities .01, family support .57, peer support .04; and for parent(s)/carer(s) employment status – facilities .02, family support .54, peer support .09. These results confirm that family support is consistently, and by a substantial margin, the most important mediator, followed by access (for the area-based SEIFA measure) or peer support (for the parent/carer-based measures).

### Moderation analysis

The chi-square test results in Table 1 for the four subsamples show very similar patterns for Year 7 and metropolitan subsamples, but less evidence of relationships for the Year 11 and regional/rural subsamples., particularly the relationship of club membership to measures of SES and access, While this is to some degree an artefact of the smaller numbers in the latter subsamples, an examination of the patterns of proportions in the cells for each crosstabulation (not shown) indicates that the associations, while present, are generally weaker in the Year 11 and regional/rural subsamples.

Moderated mediation analyses (results not shown) produced broadly similar results for Year 7 and metropolitan subsamples. For the regional/rural subsample, all relationships were much weaker and no mediation was established. For the Year 11 subsample, peer support was a relatively stronger mediator of parent/carer-based measures, and access was a relatively stronger mediator of the area-based SEIFA measure, than for the other subsamples. Furthermore, uniquely among all of the analyses, family support did not mediate the effect of the SEIFA measure for the Year 11 subsample.

In summary, both chi-square tests and mediation analysis indicated stronger relationships in metropolitan areas than in rural/regional areas between SES and sports club membership, mediated most strongly by family support and to a lesser degree by access and peer support. There was also some evidence that the relationships are stronger at Year 7 level than at Year 11 level, and that the patterns of mediation differ for the two age groups.

## Discussion

Regular participation in moderate to vigorous PA is imperative for good health. It is therefore important to understand how to facilitate participation. There is much research on the determinants of general levels of PA. However there are many different forms and contexts of PA participation and we need to understand what influences these behaviours so that strategies can be developed to increase participation in each of these specific types of PA. Club sport participation during adolescence is one context of PA participation that can positively influence PA in adulthood [59,60], and lay the foundation for fundamental motor skills [61] and competence [62] as well as providing the benefits of social interactions [63] and enhancing self-esteem [64]. This study investigated adolescent girls in two age ranges living in two types of geographical location, and found that club sport participation was a popular form of leisure time PA, particularly among younger girls and particularly in rural and regional areas. The study further investigated the relationship between club sport and household- and area-level characteristics including indicators of SES, access to facilities, and support by family and peers.

Little is known about how family structure influences PA behaviours. It has been suggested that the relationship between family structure (single v double v stepfamily) and children’s activities appears to be stronger for sedentary activities than for physical activities [53]. Furthermore, children in two-parent families reportedly have more opportunities to engage not only in activities as individuals, but also in joint activities with their parents [53]. In our study, sports club members were more likely to live with two parents, with or without siblings, but this applied predominantly to non-metropolitan adolescents and to the Year 7 cohort, and not to metropolitan adolescents or to the Year 11 cohort.

In this study ethnicity and religion were significantly associated with sports club membership. Highest rates of club membership were reported amongst those with family born in Australia and who spoke English only. Furthermore, sports club members were most likely to indicate being Christian or of no religious faith. A systematic review of correlates of physical activity reported that for adolescents ethnicity was consistently related, whereby those of ethnic minority groups were less active than others [13]. It may be that different ethnic groups are associated with different levels of family support, which is a key determinant of PA behaviour amongst adolescents [65]. Broadly, ethnicity and religion are social determinants of health that affect adolescents [66]. Ethnic identity is a known risk factor for health, however the relationship between health and religion or spirituality is not well defined [66].

SES is often reported as a key determinant of PA behaviour and health in general [7,14,47]. We found that sports club membership was predominantly associated with relatively affluent Australian-born and acculturated families living in higher SES localities. A systematic review summarised the findings of people with lower SES backgrounds being less active [12]. They concluded that as long as the activities require financial payment there is a barrier for persons with lower SES, but also that there may be many other related factors within the community, including: having fewer and poorer quality recreational areas and longer distance to PA facilities; communities perceived as being less safe; a general lack of time; and lower educational levels [12].

We also found that the evidence of association between SES and club membership was stronger in metropolitan than regional/rural settings. This was particularly so at an area level, with club membership not significantly associated with SEIFA IRSAD for regional/rural adolescent girls. This may reflect the smaller scale of rural and regional communities, which makes access to facilities less dependent on SES of home postcode, as well as lower costs of participation in PA and more limited alternative recreational options in rural and regional areas [34]. Stalsberg and Pedersen (2010) also found that almost half of the papers they reviewed reported either a negative or no relationship between SES and level of PA, which may indicate that the issue of SES differences is only partly or periodically relevant [12]. Two recent studies found that people with low SES were just as likely to participate in sport and PA [16,67]. Ball and colleagues (2009) suggested that, given the well documented inverse relationship of SES with PA levels in adult samples, such disparities may only emerge after adolescence [16].

Two other commonly reported demographic measures of SES are the employment status and educational levels of parents or caregivers. We found that these factors were significantly associated with club membership for metropolitan adolescents and for the Year 7 cohort, but not for regional/rural students or the Year 11 cohort. It has been similarly reported that adolescents with one or two unemployed parents were less likely to participate in sports that those with two employed parents [17]. A review of participation in PA for children and adolescents reported that mothers’ education level was a factor affecting participation for their children, as was family income [68]. However Ball and colleagues (2009) did not find significant evidence of influence of maternal education either cross-sectionally or longitudinally predictive of children’s PA behaviours [16].

Proximity to facilities is commonly reported to be positively associated with participation in PA [14,31,33,69]. However Grow and colleagues (2008) investigated facility location in more detail and concluded that for adolescents, proximity was not related to frequency of use of indoor recreation facilities, basketball courts and other playing fields/courts [32]. Perceived availability of sports facilities and parks has been shown to be significantly associated with engagement of adolescents in sports [70], however the agreement between objective and perceived availability of parks, sports facilities, bicycle lanes and sidewalks was low [70]. No associations were found between objectively assessed availability of sports facilities and parks and PA [70]. In a US study of adolescent girls, perceptions about both individual facilities and the total number of facilities were associated with increased PA [71]. In an Australian study, the environment – presence of public open spaces in the neighbourhood – played a smaller role than did psychosocial factors [7]. However, a recent systematic review of reviews of environmental correlates of PA amongst children and adolescents found that physical activity was more consistently related to school and neighbourhood characteristics than to interpersonal and societal environments [24]. As defined in the present study, access encompassed both knowledge regarding the existence and location of facilities and perceptions of their ability to get there, either by themselves or with the assistance of an adult for transport. We found that in general, club members reported significantly better access to club based activities, but this was not the case for geographically widespread facilities/venues such as beach, bike lanes, lakes, rivers and creeks, parks and playgrounds. Level of access was a more significant determinant for the Year 7 cohort.

Many studies confirm the importance of social support from parents regarding adolescents’ PA [22,23,72]. This is especially so in younger years and regardless of the child’s gender [68], but when a relationship is found between the mother’s support and their child’s PA, it is more often associated with girls [68]. However this correlation is not always reported. In a study of adolescents and young adults there was no significant differences in perceived parental support relating to PA behaviour [21,24,26]. Paternal care for staying fit and exercising was, however, related to increased MVPA for older females [21]. In our study family support was a significant predictor of club membership. This support included travel, encouragement, playing with their girls, watching, and praising their involvement in sport and physical activity. Family support was a significant factor for both Year 7 and Year 11 girls. This is in contrast to another Australian study which found family support was a positive influence for Year 10’s but not for the younger Year 7’s [20]. It has been stated that this support differs across adolescence, with peers having greater influence on older adolescents when they are more autonomous and not as reliant on parents for travel [23]. These researchers also found that younger adolescents receive more praise and joint participation from parents [23].

Many studies have found the importance of peer influence on out-of school PA behaviours [26], or general MVPA levels [20]. In our study, peer support was significantly related to club membership. Furthermore, we found that negative peer interaction i.e. teasing for not being good at the activity was not a significant barrier to club membership.

With regard to mediation of the relationships between measures of SES (SEIFA IRSAD of home postcode, education level and employment status of parents/caregivers) and club membership, we found that access to facilities, family support and peer support were all mediators of the associations between particular aspects of SES and club membership. The most consistent and by far the strongest mediator, across the three dimensions of SES we examined, was the interpersonal factor of family support. That is, the level of family support is the most crucial channel through which the effects of SES influenced the adolescent girls’ participation in club sport. With regard to the other interpersonal factor we examined, peer support, we found that the adolescent girls’ perceptions of peer support were more influenced by the employment status of their parents/caregivers than by either parental education level or neighbourhood SES, as measured by postcode SEIFA IRSAD. This may be because of the more pervasive and immediate effects of parental employment status on adolescents’ daily lives. Be that as it may, peer support partially mediated only the link between parental employment status and club membership. Access to facilities, whilst it has an interpersonal aspect to the extent that adolescents depend on parents/caregivers for transport, is primarily an environmental determinant in terms of geography and proximity to facilities. As such, it was found to be an important mediator for neighbourhood SES, as measured by postcode SEIFA IRSAD, but was not a significant mediator for household-based measures of SES. In comparison, Cerin and Leslie (2008) also reported that differences in social support, as well as perceived benefits and self-efficacy, were largely responsible for the observed individual- and area-level SES differences in regular participation in leisure-time PA [7]. In contrast, in the Trial of Activity in Adolescent Girls (TAAG) study, environmental factors appeared to be the strongest mediators (compared to behavioural and psychosocial) between the intervention and the activity level [26]. The environmental factors mainly related to issues around transport to and from activities [26]. The focus of the TAAG study more on the school and community than the home environment may explain some of the differences between study results. Furthermore, the issues reported around access may also be related to lack of support from family in relation to travel.

With regard to moderation of effects, we found stronger relationships in metropolitan areas than in rural/regional areas between SES (both area- and household-level indicators) and sports club membership. It is known that postcodes in rural areas have more heterogeneous SES profiles than those in urban areas [73]and it may be that rural/regional communities have more heterogeneous SES profiles than do metropolitan (suburban) communities at a similar ‘social scale’ (i.e. the scale over which interactions between adolescents take place), and hence that in rural/regional communities behaviours such as sports club membership are likely to be influenced by groups (peers, peers’ parents, school communities) with a broader range of SES characteristics. There was also some evidence that the relationships are stronger at Year 7 level than at Year 11 level. This too may be explained by increased autonomy and mobility, and consequential exposure to a wider range of influences, in later adolescence.

In summary, we found stronger relationships between sports club membership and SES for the metropolitan rather than rural females. These relationships were mediated mostly by family support and less by access and peer support. These results concur with very recent literature which reports that the family environment, including provision of equipment, financial, logistic and emotional support and parental modelling are positively associated with sport participation [49].

### Assumptions and limitations

The mediation models in this study, as with all such models, are framed in causal terms, with the intervening variables — access to facilities, family support and peer support — mediating the effect of SES on sports club membership. In general one should be cautious about attributing causation in cross-sectional studies. However, in this study the converse — that sports club membership influences SES — is considered unlikely.

The relatively high prevalence of sports club membership in the sample suggests self-selection bias, with the more active girls more likely to participate in the study. However, even if such bias were present, this would not of itself threaten the validity of the mediation analysis.

## Conclusions

The results of this study indicate that participation in club sport among both younger and older adolescent girls is strongly positively associated with the SES of both their neighbourhoods and their households, particularly in metropolitan areas. These associations are most strongly mediated by family support and by access to facilities. Consequently, in order to facilitate and promote greater participation in club sport among adolescent girls from low SES neighbourhoods and households, strategies should target modifiable determinants such as facility access and parental support. This will involve improving access to sports facilities, and promoting, encouraging, and assisting parents to provide support for their daughters’ participation in sports clubs. Strategies could include an educational social marketing campaign targeting parents that emphasises the positive health benefits of sports club involvement and the key role that parents play in promoting and facilitating this involvement. An educational program and resources outlining these sports club benefits to parents and adolescents could also be based at secondary schools. Sport and recreation facility planning at the local and state government level also needs to be sensitive to the needs of women and girls.

## Abbreviations

DV: Dependent variable; EV: Explanatory variable; IRSAD: Index of relative socio-economic advantage and disadvantage; MVPA: Moderate or vigorous physical activity; PA: Physical activity; SEIFA: Socio-economic Indices for areas; SES: Socio-economic status; TAAG: Trial of activity in adolescent girls.

## Competing interests

The authors declare that they have no competing interests.

## Authors’ contributions

RE contributed to the study design, questionnaire design, collection of data, interpretation of results, manuscript conceptualisation and preparation. JH contributed to the study design, questionnaire design, data management, statistical analysis and interpretation, manuscript conceptualisation and preparation. MC contributed to the study design, questionnaire design, critical review of the manuscript. CS contributed to the study design, questionnaire design, critical review of the manuscript. WP contributed to the study design, questionnaire design, interpretation of results, manuscript conceptualisation and manuscript preparation. All authors have read and approved the final manuscript.
